# Supplementary figures and images for: Screening and Identification of Drought-Tolerant Genes in Tomato (Solanum lycopersicum L.) Based on RNA-Seq Analysis
Source: Plants (Basel). 2025 May 14;14(10):1471. doi: 10.3390/plants14101471 (PMC12114640; doi:10.3390/plants14101471)

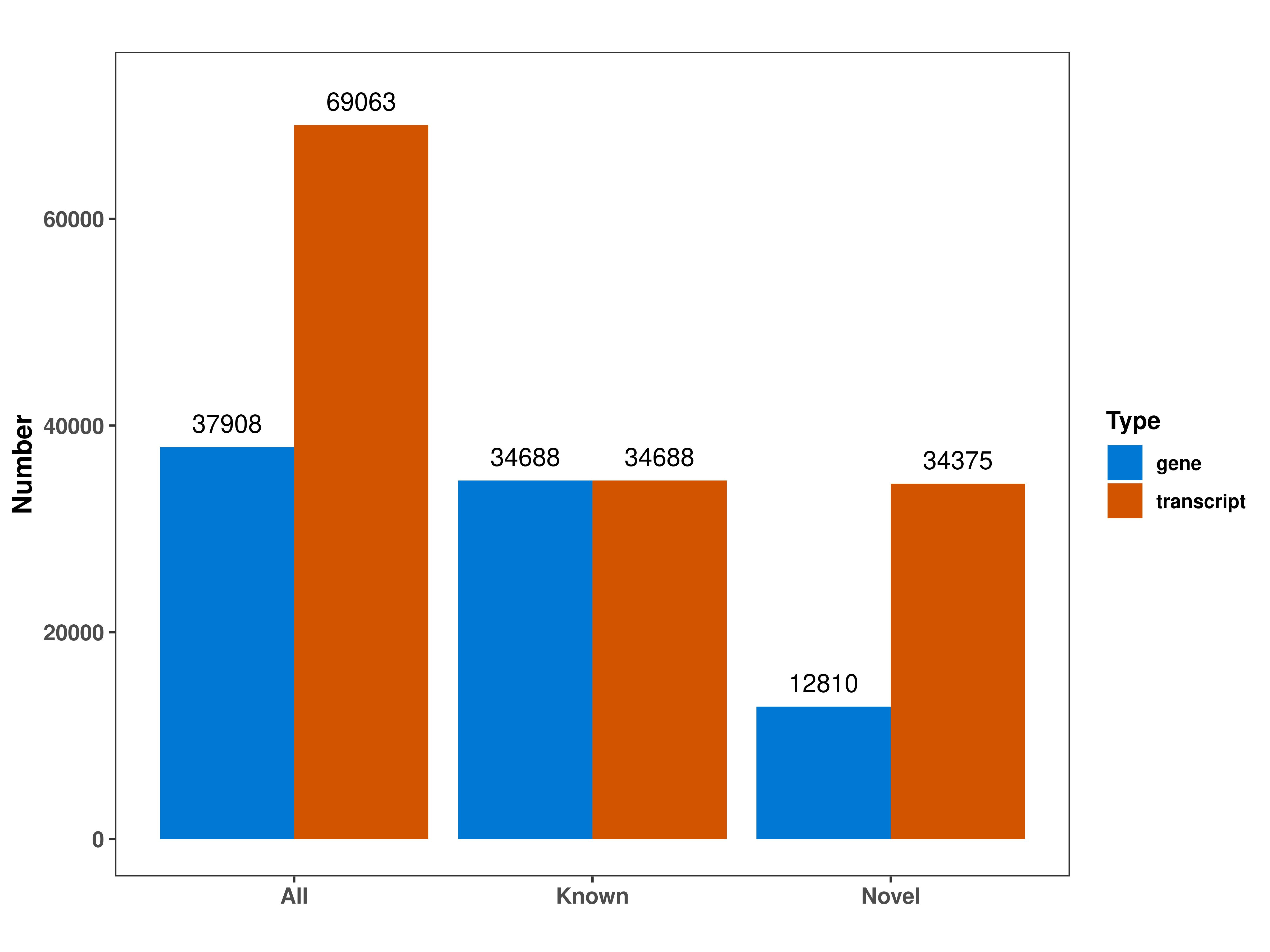

Supplement: Supplementary file 1 [file plants-14-01471-s001.zip › Figure S1.jpg]
